# Supplementary figures and images for: Machine Learning Heuristics on Gingivobuccal Cancer Gene Datasets Reveals Key Candidate Attributes for Prognosis
Source: Genes (Basel). 2022 Dec 16;13(12):2379. doi: 10.3390/genes13122379 (PMC9777687; doi:10.3390/genes13122379)

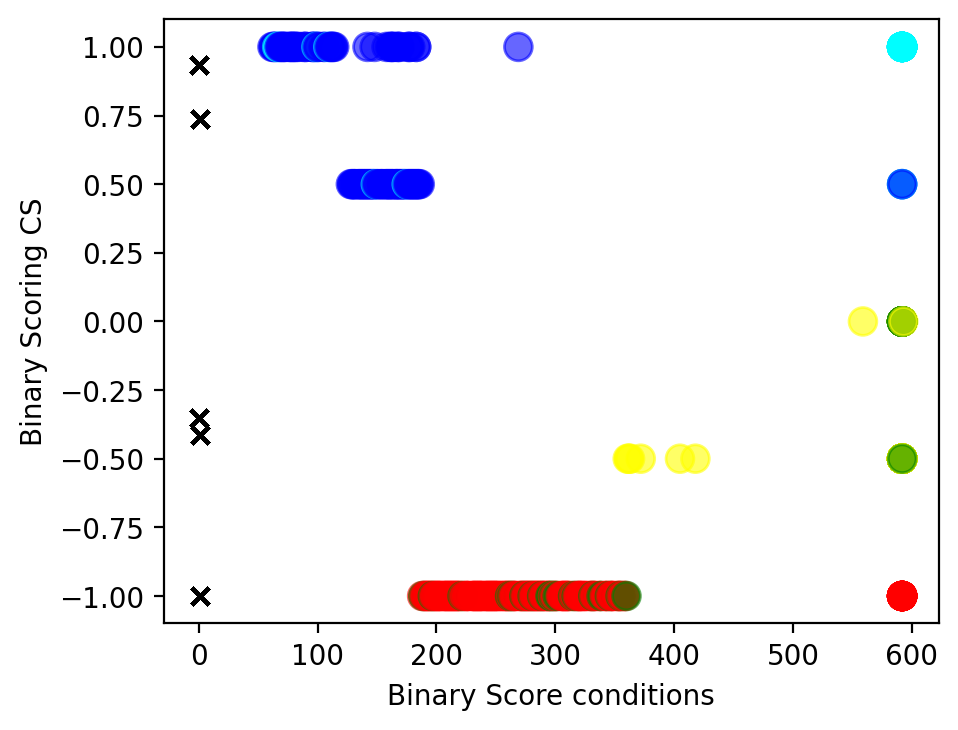

Supplement: Supplementary file 1 [file genes-13-02379-s001.zip › Figure S1/Binary Score conditions.png]

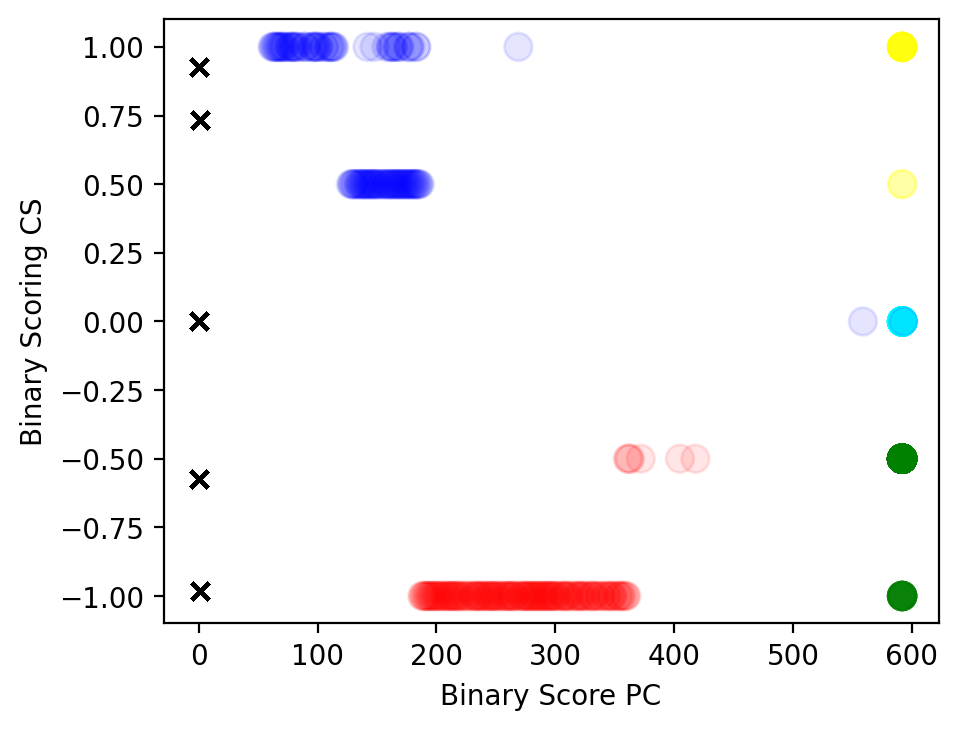

Supplement: Supplementary file 1 [file genes-13-02379-s001.zip › Figure S1/BinaryScore PC.png]

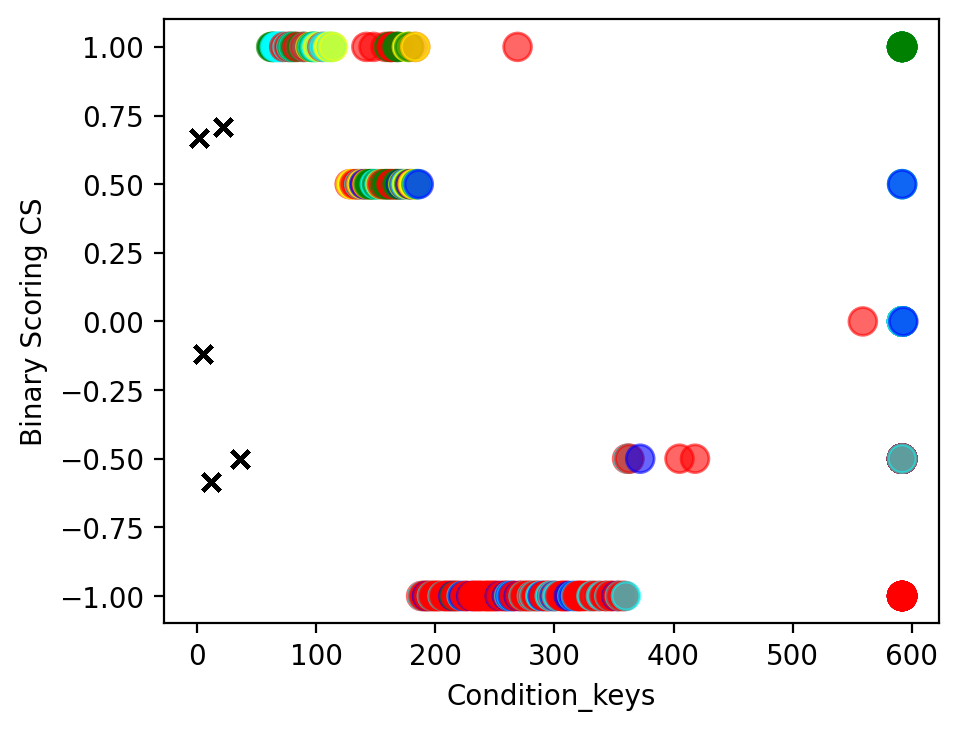

Supplement: Supplementary file 1 [file genes-13-02379-s001.zip › Figure S1/Condition_keys.png]

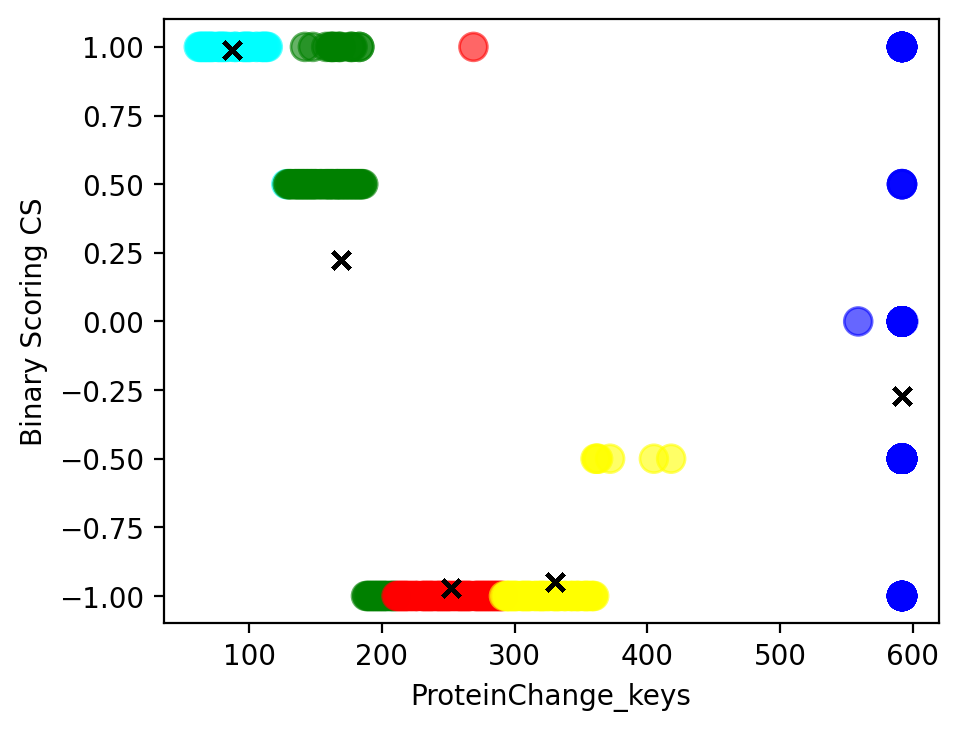

Supplement: Supplementary file 1 [file genes-13-02379-s001.zip › Figure S1/ProteinChange_Keys.png]
